# Automated Quantification of Neuropad Improves Its Diagnostic Ability in Patients with Diabetic Neuropathy

**DOI:** 10.1155/2015/847854

**Published:** 2015-05-12

**Authors:** Georgios Ponirakis, Hassan Fadavi, Ioannis N. Petropoulos, Shazli Azmi, Maryam Ferdousi, Mohammad A. Dabbah, Ahmad Kheyami, Uazman Alam, Omar Asghar, Andrew Marshall, Mitra Tavakoli, Ahmed Al-Ahmar, Saad Javed, Maria Jeziorska, Rayaz A. Malik

**Affiliations:** ^1^Research Division, Weill Cornell Medical College in Qatar, Qatar Foundation, P.O. Box 24144, Education City, Doha, Qatar; ^2^Institute of Human Development, Centre for Endocrinology & Diabetes, Faculty of Medical and Human Sciences, University of Manchester and NIHR/Wellcome Trust Clinical Research Facility, Manchester M13 9NT, UK; ^3^Roke Manor Research Ltd, Old Salisbury Lane, Romsey, Hampshire SO51 0ZN, UK

## Abstract

Neuropad is currently a categorical visual screening test that identifies diabetic patients at risk of foot ulceration. The diagnostic performance of Neuropad was compared between the categorical and continuous (image-analysis (Sudometrics)) outputs to diagnose diabetic peripheral neuropathy (DPN). 110 subjects with type 1 and 2 diabetes underwent assessment with Neuropad, Neuropathy Disability Score (NDS), peroneal motor nerve conduction velocity (PMNCV), sural nerve action potential (SNAP), Deep Breathing-Heart Rate Variability (DB-HRV), intraepidermal nerve fibre density (IENFD), and corneal confocal microscopy (CCM). 46/110 patients had DPN according to the Toronto consensus. The continuous output displayed high sensitivity and specificity for DB-HRV (91%, 83%), CNFD (88%, 78%), and SNAP (88%, 83%), whereas the categorical output showed high sensitivity but low specificity. The optimal cut-off points were 90% for the detection of autonomic dysfunction (DB-HRV) and 80% for small fibre neuropathy (CNFD). The diagnostic efficacy of the continuous Neuropad output for abnormal DB-HRV (AUC: 91%, *P* = 0.0003) and CNFD (AUC: 82%, *P* = 0.01) was better than for PMNCV (AUC: 60%). The categorical output showed no significant difference in diagnostic efficacy for these same measures. An image analysis algorithm generating a continuous output (Sudometrics) improved the diagnostic ability of Neuropad, particularly in detecting autonomic and small fibre neuropathy.

## 1. Introduction

Diabetic peripheral neuropathy (DPN) is a progressive manifestation of diabetes with length-dependent and symmetrical damage of nerve fibers [1]. It is of critical importance to detect early neuropathy in the distal small nerve fibres in order to predict and prevent progressive morbidity that may involve pain, imbalance, foot deformities, ulceration, and amputation [2, 3]. However, early subclinical neuropathy cannot be diagnosed with currently endorsed clinical tests such as the 10 g monofilament or Neuropathy Disability Score (NDS) [4]. These methods primarily identify patients with established DPN who are already at high risk of foot ulceration. Given that small fibre neuropathy (SFN) is the earliest manifestation of DPN and plays a crucial role in the aetiopathogenesis of foot ulceration due to loss of pain sensation, anhidrosis, and deranged tissue blood flow, a screening test should ideally evaluate these fibres.

Several tests for evaluating SFN have been established in clinical practice and research settings but each has their own limitations. Warm perception threshold (WPT) testing detects nerve dysfunction but is expensive (£20K) and limited by the need for subjective responses and variable reproducibility. Deep Breathing-Heart Rate Variability (DB-HRV) detects autonomic nerve dysfunction but again requires expensive equipment (£10K) and patient cooperation with potential confounders such as medication and caffeine consumption [2]. Intraepidermal nerve fibre density (IENFD) is the gold standard for assessing small nerve fibre morphology from skin biopsies but is invasive and painful [5]. Corneal confocal microscopy (CCM) represents an alternative imaging technique which is a noninvasive alternative to skin biopsy [6]. It has been validated for assessing early small fibre damage and repair but requires expensive equipment and trained staff to perform the test. Sudomotor abnormalities can be detected using skin biopsy or measures of sweating using the QSART [7] or Sudoscan devices [8]. Neuropad measures sweat production based on a colour change in a cobalt II compound from blue to pink to produce a categorical output but has a moderate diagnostic performance for DPN [9–13]. Neuropad specificity for large fibre neuropathy is low (50–64%), whereas for small fibre neuropathy SFN it is much higher (80%) [14]. Other studies have also reported low specificity (45–67.2%) for large fibre neuropathy measures such as NDS and Vibration Perception Threshold [12, 15–17].

The diagnostic validity of the Neuropad response has been tested primarily for categorical [10, 11, 15–17] rather than a continuous output [14]. Hence for the categorical output there are only three possible outcomes: normal, intermediate, or abnormal. Whilst this provides an output, which is simple to interpret by both the patient and clinician, it lacks discrimination for minor worsening or improvement. To address this we have previously proposed a continuous output expressed as a percentage colour change determined visually [14], but this is subjective with a coefficient of repeatability for intra- and interobserver variability of 0.3 and 0.4, respectively. This argues for the development of image analysis software to rapidly and consistently grade the colour change to a percentage output, enabling a continuous quantitative and completely reproducible measure of sudomotor small fibre dysfunction.

In the present study we have tested the diagnostic ability of Sudometrics software, which can quantify the Neuropad response in a range from 0 to 100% against the established categorical output for measures of SFN and LFN.

## 2. Research Design and Methods

The participants in the study were recruited from the Manchester Diabetes Centre, Manchester Royal Infirmary in Manchester, UK. The study was performed at the Wellcome Trust Clinical Research Facility/NIHR from September 3, 2012, to May 30, 2014, involving 110 subjects with diabetes mellitus (DM) (84 type 1 DM and 26 type 2 DM) with an average age of 53 ± 13 years. We estimated that the minimum sample required to detect significant difference in Neuropad response between the group with DPN and without DPN was 68 participants by means of an unpaired *t*-test and with a power of 95%. Exclusion criteria included history of neuropathy due to nondiabetic cause and corneal trauma or surgery. This study was approved by the Local Research Ethics committee and all patients gave informed consent to take part in the study. The research adhered to the tenets of the Declaration of Helsinki.

### 2.1. Demographic Measures

All study participants underwent assessment of their glycated haemoglobin (HbA1c), body mass index (BMI), systolic and diastolic blood pressure, cholesterol, and triglycerides.

### 2.2. Functional Tests of Small Nerve Fibres

The function of the small cholinergic and adrenergic nerves that regulate sweating in the feet was measured by Neuropad (miro Verbandstoffe, Wiehl-Drabenderhöhe, Germany) [18]. The plaster was applied to the plantar aspect of the 1st metatarsal head after callus removal and removed after 10 minutes. Immediately after removal, the plaster was scanned in high resolution 600 dpi by the Fujitsu FI-60F fast flatbed passport scanner (Response Technical Services Ltd, Surrey, UK). The percentage colour change in pink over the whole area of Neuropad was estimated by Sudometrics. The algorithm is based on the intensity-level analysis of the pink colour. Once the area of Neuropad is segmented using a variable threshold a colour histogram is computed and the pink percentage is extracted as a metric. A morphological set operation is conducted to remove background noise and better define the pad area. Sudometrics is available to all potential collaborators solely for research purposes (non-for-profit/noncommercial). It is protected by the University of Manchester in the form of license agreement which can be requested online (http://www.click2go.umip.com/i/software/Biomedical_Software/Sudometrics.html).

Cardiac autonomic function was measured using the ANX 3.0 autonomic nervous system monitoring device (ANSAR Medical Technologies Inc., Philadelphia, US) [19]. Deep Breathing-Heart Rate Variability (DB-HRV) was assessed by R-R interval variation via surface electrodes. DB-HRV was recorded over 1 min at a frequency of 6 breaths/min.

Thermal discrimination threshold testing was undertaken on the dorsum of the left foot using the MEDOC* TSA II* (Medoc Ltd., Ramat Yishai 30095, Israel) and method of limits [20].

### 2.3. Structural Tests of Small Nerve Fibres

#### 2.3.1. Corneal Confocal Microscopy

Patients underwent examination with the Heidelberg Retina Tomograph (HRT III RCM)* in vivo *corneal confocal microscope (IVCCM) (Heidelberg Engineering GmbH, Heidelberg, Germany) using our established methodology [21]. The section mode enables manual acquisition and storage of single images of the central cornea with a lateral resolution of approximately 2 *μ*m/pixel and final image size of 400 × 400 pixels of the subbasal nerve plexus. Corneal Nerve Fibre Density (CNFD), the total number of nerve fibres (no./mm^2^), Corneal Nerve Branch Density (CNBD), the total number of nerve branches (no./mm^2^), and Corneal Nerve Fibre Length (CNFL), the total length of all nerve fibres and branches (mm/mm^2^) within the area of cornea captured by the image were quantified from ~5 adjacent images/subject, using purpose built manual image analysis software called CCMetrics [21]. CCMetrics is available to all potential collaborators solely for research purposes (non-for-profit/noncommercial). It is protected by the University of Manchester in the form of license agreement which can be requested online (http://www.human-development.manchester.ac.uk/ena/ACCMetricsuserinstructions#Researchlicenceagreement).

#### 2.3.2. Intraepidermal Nerve Fibre Density

A 3 mm punch skin biopsy was taken from the dorsum of the foot under 1% lidocaine local anaesthesia. Skin samples were immediately fixed in 4% (wt/vol.) paraformaldehyde for 24 h and then cryoprotected in sucrose for 18 h and cut into 50 *μ*m thick sections. Immunohistochemistry was performed as previously described [9]. An image analysis camera AxioCam MRc (Ziess, Germany) and Leica QWin Standard V2.4 (Leica Microsystem Imaging, Cambridge, UK) were used to quantify intraepidermal nerve fibre density (IENFD), which is the total number of nerve fibres per millimeter length of epidermis (no./mm).

### 2.4. Neuropathy Assessments

All patients underwent an assessment of neuropathy based on a standard protocol including Neuropathy Disability Score (NDS) to classify participants into without (NDS 0–2) and with (NDS 3–10) neuropathy [4, 22]. Quantitative sensory testing included an assessment of Vibration Perception Threshold (VPT), measured using a Neurothesiometer (Horwell, Scientific Laboratory Supplies, Wilford, Nottingham, UK) and warm perception thresholds (WPT) using the method of limits with the MEDOC* TSA II* (Medoc Ltd., Ramat Yishai 30095, Israel) on the dorsum of the left foot. Electrodiagnostic studies were undertaken using a Dantec “Keypoint” system (Dantec Dynamics Ltd., Bristol, UK) equipped with a DISA temperature regulator to keep limb temperature constantly between 32 and 35°C. Sural nerve conduction velocity (SNCV), sural sensory nerve action potential (SNAP), peroneal motor nerve conduction velocity (PMNCV), and peroneal motor nerve action potential (PMNAP) were assessed in the right lower limb by a consultant neurophysiologist.

### 2.5. Study Definition of Diabetic Peripheral Neuropathy

The Toronto Diabetic Neuropathy Expert group recommendation was followed to define DPN: (a) abnormal PMNCV (<42 m/s) and (b) abnormal symptoms or signs of neuropathy, NDS (>2) [23].

To define an abnormal result for each of the measures of neuropathy we have used a mean ± 2 SD cut-off based on our control population (*n* = 104).

### 2.6. Statistical Analysis

Statistical analysis was performed using StatsDirect statistical software, version 2.7.9. We examined the distribution of the data by means of relevant histograms and the Shapiro-Wilk test. All data were expressed as median (5th percentile, 95th percentile). Mann-Whitney *U* test was performed to analyse differences between the medians. A *P* value <0.05 was considered statistically significant.

Receiver operating characteristic (ROC) curve analysis was used to compare the diagnostic accuracy of Neuropad against measures of large and small nerve fibre damage. ROC curve analysis established the area under the curve (AUC) to determine the optimal sensitivity and specificity of the Neuropad test. Statistical difference between two ROC curves was expressed in *P* value as described by Hanley and McNeil [24].

## 3. Results and Discussion

### 3.1. Clinical Data

Of the 110 subjects with diabetes, 46 were diagnosed with and 64 without diabetic peripheral neuropathy (DPN). The demographic and clinical characteristics of the participants with and without DPN are presented in Table 1. BMI, diastolic blood pressure, cholesterol, and triglyceride levels did not differ between the two groups, but HbA1c (*P* = 0.006), age (*P* < 0.0001), duration of diabetes (*P* < 0.0001), and systolic blood pressure (*P* = 0.004) were significantly higher in those with DPN. The group with DPN had a significantly higher Neuropathy Disability Score (NDS) and Vibration Perception Threshold (VPT) and significantly lower sural sensory nerve action potential (SNAP), sural nerve conduction velocity (SNCV), peroneal motor nerve conduction velocity (PMNCV), and peroneal motor nerve action potential (PMNAP) (*P* < 0.0001 for all comparisons). NDS and nerve conduction parameters had substantial variability indicating that the group with DPN had a wide severity of neuropathy. Similarly the DPN group had a significantly lower Neuropad response (*P* = 0.01), intraepidermal nerve fibre density (IENFD) (*P* < 0.0001), CNFD (*P* = 0.005), CNFL (*P* = 0.03), and Deep Breathing-Heart Rate Variability (DB-HRV) (*P* = 0.002) with a significantly higher warm perception threshold (WPT) (*P* < 0.0001).

### 3.2. Neuropad Diagnostic Performance for Diabetic Peripheral Neuropathy

The continuous output was generated using image analysis software (Sudometrics) to enable rapid and consistent quantification of the Neuropad response. The evaluation of colour change in percentage was completely reproducible with a coefficient of repeatability of 1. The diagnostic performance between the continuous and the categorical output is presented in Table 2. The continuous output displayed high sensitivity and specificity for DB-HRV (91%, 83%), CNFD (88%, 78%), and SNAP (88%, 83%) and high sensitivity with moderate specificity for CNBD (83%, 72%), CNFL (89%, 75%), and VPT (80%, 71%), respectively. For the categorical output the sensitivity was equally high but the specificity was lower for DB-HRV (82%, 59%), CNFD (89%, 63%), CNBD (100%, 47%), CNFL (90%, 50%), SNAP (100%, 55%), and VPT (70%, 57%), respectively. This echoes our recent findings [14] that a continuous Neuropad output has high sensitivity (83%) and specificity (80%) for detecting structural small fibre damage.

### 3.3. Small versus Large Fibre Neuropathy

Early subclinical SFN precedes large fibre impairment with numbness and foot ulceration [1]. Currently, advocated clinical tests such as the 10 g monofilament or Neuropathy Disability Score (NDS) cannot detect patients with early neuropathy [4]. Neuropad has been promoted as an inexpensive, practical, first-line diagnostic screening test for subclinical SFN [9–11].

This study highlights the parameters for which the Neuropad is most useful for the diagnosis of early DPN. Furthermore, it clearly shows that a continuous output for Neuropad significantly improves its diagnostic ability to detect autonomic and SFN than measures of large fibre neuropathy. The AUC for DB-HRV (91%) was significantly larger than for NDS (67%, *P* = 0.001), VPT (75%, *P* = 0.02), SNCV (62%, *P* = 0.0009), PMNAP (63%, *P* = 0.003), and PMNCV (60%, *P* = 0.0003). Similarly, the AUC for CNFD (82%) was significantly larger than for NDS (67%, *P* = 0.05), SNCV (62%, *P* = 0.02), PMNAP (63%, *P* = 0.04), and PMNCV (60%, *P* = 0.01). The AUC for CNFL (80%) was significantly larger than for PMNCV (60%, *P* = 0.05). Unlike the continuous output, the categorical output showed no significant difference between small and large fibre damage. Indeed, we have previously shown that Neuropad correlates better with heart rate variability than NDS [9]. Of relevance, previously, CNBD, CNFD, and CNFL have also been shown to correlate highly significantly with heart rate variability [25].

**Figure 1:**
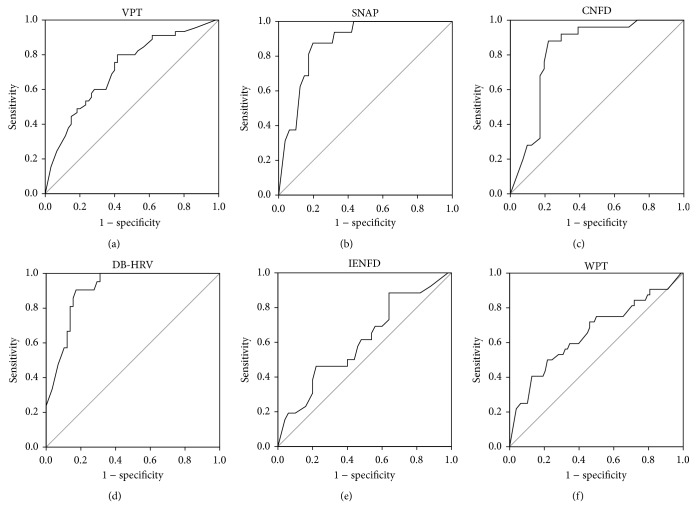
ROC curve analysis was used to compare the diagnostic accuracy of Neuropad in detecting large with small nerve fibre damage (black line). The grey line is the null value of the ROC curve. The AUC for large nerve fibre damage was (a) 75% for Vibration Perception Threshold (VPT) (SE: 0.049, 95% CI 0.53–0.97) and (b) 86% for sural sensory nerve action potential (SNAP) (SE: 0.04, 95% CI 0.42–1). The AUC for small nerve fibre damage was (c) 82% for Corneal Nerve Fibre Density (CNFD) (SE: 0.053, 95% CI 0.48–1), (d) 91% for Deep Breathing-Heart Rate Variability (DB-HRV) (SE: 0.031, 95% CI 0.49–1), (e) 63% for intraepidermal nerve fibre density (IENFD) (SE: 0.068, 95% CI 0.45–0.81), and (f) 69% for warm perception thresholds (WPT) (SE: 0.05, 95% CI 0.49–0.88).

Since Neuropad is a diagnostic screening test for sudomotor function and hence, for SFN, we propose that the optimal cut-off point for small fibre dysfunction is 90% and for small fibre structural damage it is 80%, based on DB-HRV and corneal nerve morphology (CNBD, CNFD, and CNFL), respectively. The optimal cut-off point is the percentage of Neuropad colour change to pink defining normal from abnormal. Our current data are consistent with previous studies showing a strong association between Neuropad and autonomic neuropathy [19] as well as small fibre neuropathy assessed using corneal confocal microscopy [26]. However, interestingly there is considerable variability for the diagnostic ability (AUC) of a continuous Neuropad output amongst the different measures of small and large fibre neuropathy. Thus whilst there is a good diagnostic ability for SNAP (86%), it was only moderate for SNCV (62%), PMNCV (60%), and PMNAP (63%) as well as VPT (75%).  Figure 1 shows that Neuropad exhibited a higher AUC for DB-HRV (91%), rather than WPT (69%), supporting the expected association with autonomic rather than somatic small fibre dysfunction. Rather surprisingly the diagnostic performance of Neuropad to identify structural pathology of the small fibres was better for CNFD (82%), CNBD (79%), and CNFL (80%) than IENFD (63%). These different associations were similar but not as strong with the categorical output (Table 2).

This study has several strengths and limitations. We utilised a detailed and comprehensive evaluation of a wide range of gold standard techniques to quantify diabetic neuropathy, which enabled us to dissect and define the diagnostic ability of the Neuropad in a large cohort of individuals with type 1 and 2 diabetes. Furthermore, unlike all previous studies [9, 12, 13], including our recent study [14] the estimation of colour change for the Neuropad has been categorical or subjective and hence more liable to error. In the current study we have utilized an image analysis algorithm (Sudometrics), to provide a continuous and completely reproducible output. DPN was of course diagnosed using the Toronto consensus, which is large fibre weighted and incorporation of a small fibre abnormality as per a previous consensus may well have further improved the diagnostic ability of Neuropad as observed with the much higher AUCs with measures of small fibre neuropathy [3]. We were not able to account for the potential confounding effect of age, diabetes duration, HbA1c, systolic blood pressure, and BMI on the relationship that Neuropad has with small and large fibre damage because of their inherent influence on DPN. We believe our data support the use of a continuous output for Neuropad as a diagnostic test for DPN, but a longitudinal study is required to assess the predictive and hence prognostic ability of this simply administered test. Furthermore, it is important to note that the Sudometrics output requires a high resolution scanner and image analysis software, which may be deemed cumbersome and takes from the simple visual output provided by Neuropad to the patient and clinician. However, we would propose that given the advances in technology this whole process could easily be incorporated into a mobile App, which the manufacturer of Neuropad should explore.

## 4. Conclusions

In conclusion, the current study shows that the diagnostic efficacy of Neuropad can be considerably enhanced using Sudometrics, an automated continuous output as opposed to the categorical output, particularly for evaluating SFN. Given the advances in mobile technology, this merits exploration to develop a user friendly App for this purpose.

## Figures and Tables

**Table 1:** Comparison of clinical data of study participants with type 1 and 2 diabetes according to the presence or absence of neuropathy defined by the Toronto criteria. Data are medians (5th percentile, 95th percentile), *P* values are derived from a Mann-Whitney *U* test.

Variables	Diabetes without neuropathy	Diabetes with neuropathy	*P* value
Demographic measures			
*n*	64	46	
Age	45 (21, 71)	62 (44, 75)	<0.0001
Diabetes duration (years)	19 (3, 48)	40 (8, 58)	<0.0001
Gender (male/female)	39/25	33/13	
Type of diabetes (I/II)	52/12	32/14	
HbA1c % [mmol/mol]	7.6 (6.9, 8.4) [60 (51, 68)]	8.6 (8, 9.2) [69 (59, 77)]	0.006
BMI (kg/m^2^)	26.0 (21, 38)	29.0 (21, 38)	0.09
Systolic BP (mmHg)	123 (99, 154)	140 (108, 169)	0.004
Diastolic BP (mmHg)	67 (56, 78)	67 (56, 83)	0.9
Cholesterol (mmol/L)	4.2 (2.9, 6.9)	3.8 (2.7, 6)	0.17
Triglyceride (mmol/L)	1 (0.5, 2)	1.1 (0.4, 2.9)	0.4

Large fibre assessments			
NDS	0.5 (0, 5)	5.5 (3, 10)	<0.0001
VPT (V)	6.3 (3, 19)	21.8 (8, 41)	<0.0001
SNAP (*μ*V)	13 (4.6, 28)	4.9 (0.4, 19)	<0.0001
SNCV (m/s)	43.8 (40, 51.9)	39.5 (27, 45.7)	<0.0001
PMNAP (*μ*V)	4.6 (1, 7.6)	1.8 (0.1, 5)	<0.0001
PMNCV (m/s)	44.1 (39.1, 49.1)	39.3 (19, 45)	<0.0001

Small fibre assessments			
IENFD (no./mm)	6.8 (0.5, 13.5)	3.5 (0.3, 15.3)	<0.0001
CNFD (no./mm^2^)	30.0 (15.6, 43.8)	21.4 (6.3, 36.5)	0.005
CNBD (no./mm^2^)	90.1 (21.9, 214)	62.5 (3.1, 220.3)	0.1
CNFL (mm/mm^2^)	25.0 (12.8, 33.5)	19.6 (4.6, 34.0)	0.03
DB-HRV (beats per min)	25 (6, 45)	10 (4, 39)	0.002
WPT (°C)	38.5 (34.7, 46.7)	42.0 (36.3, 49.8)	<0.0001
Neuropad (%)	61.5 (0, 99)	18.0 (0, 99)	0.01

**Table 2:** Comparing the diagnostic performance of Neuropad between the continuous and the categorical output. The evaluation was performed against large (Neuropathy Disability Score (NDS) and Vibration Perception Threshold (VPT), sural sensory nerve action potential (SNAP), sural nerve conduction velocity (SNCV), peroneal motor nerve action potential (PMNAP), and peroneal motor nerve conduction velocity (PMNCV)) and small (intraepidermal nerve fibre density (IENFD), Corneal Nerve Fibre Density (CNFD), Corneal Nerve Branch Density (CNBD), Corneal Nerve Fibre Length (CNFL), Deep Breathing-Heart Rate Variability (DB-HRV), and warm perception thresholds (WPT)) nerve fibre assessments as reference methods using ROC curve analysis.

Variables	Continuous output		Categorical output		*P* value of AUC
	AUC %	Sensitivity & Specificity %	AUC %	Sensitivity and Specificity %	
Large fibre assessments					
NDS (>2)	67	71, 58	66	69, 62	0.46
VPT (>14 V)	75	80, 71	66	70, 57	0.33
SNAP (<3 *μ*V)	86	85, 83	82	100, 55	0.35
SNCV (<43 m/s)	62	66, 61	61	61, 59	0.46
PMNAP (<2 *μ*V)	63	67, 54	61	62, 50	0.44
PMNCV (<42 m/s)	60	62, 58	57	60, 53	0.39

Small fibre assessments					
IENFD (<4 no./mm)	63	65, 54	55	56, 51	0.27
CNFD (<24 no./mm^2^)	82	88, 78	79	89, 63	0.37
CNBD (<18 no./mm^2^)	79	83, 72	71	100, 47	0.32
CNFL (<14 mm/mm^2^)	80	89, 75	71	90, 50	0.26
DB-HRV (<10 beats per min)	91	91, 83	78	82, 59	0.06
WPT (>42°C)	69	75, 60	66	69, 53	0.38
